# Cosserat Rod-Based Tendon Friction Modeling, Simulation, and Experiments for Tendon-Driven Continuum Robots

**DOI:** 10.3390/mi16030346

**Published:** 2025-03-19

**Authors:** Honghong Wang, Jingli Du, Yi Mao

**Affiliations:** 1 School of Mechano-Electronic Engineering, Xidian University, Xi’an 710071, China; 2 School of Chemical and Material Engineering, Jiangnan University, Wuxi 214122, China; maoyi@stu.jiangnan.edu.cn

**Keywords:** cosserat rod, tendon-driven continuum robots, continuum robot, tendon friction, tendon routing

## Abstract

Traditional tendon-driven continuum robot (TDCR) models based on Cosserat rod theory often assume that tendon tension is a continuous wrench along the backbone. However, this assumption overlooks critical factors, including the discrete arrangement of disks, the segmented configuration of tensioned tendons, and the friction between tendons and guide holes. Additionally, tendon forces are not continuous but discrete, concentrated wrenches, with the frictional force magnitude and direction varying based on the TDCR’s bending configuration. We propose a TDCR modeling method that integrates Cosserat rod theory with a finite element approach to address these limitations. We construct a Cosserat rod model for the robot’s backbone, discretize the tendon geometry using the finite element method (FEM), and incorporate friction modeling between tendons and guide holes. Furthermore, we introduce an algorithm to determine the direction of friction forces, enhancing modeling accuracy. This approach results in a more realistic and comprehensive mathematical representation of TDCR behavior. Numerical simulations under various tendon-routing scenarios are conducted and compared with classical TDCR models. The results indicate that our friction-inclusive model improves accuracy, yielding an average configuration deviation of only 0.3% across different tendon routings. Experimental validation further confirms the model’s accuracy and robustness.

## 1. Introduction

Over the past two decades, continuum robots have made remarkable strides in modeling, control, and applications. Due to their flexible materials and tendon-actuated mechanisms, TDCRs have demonstrated exceptional adaptability in confined, complex, and unstructured environments. Consequently, they have been widely employed in diverse fields, including minimally invasive surgery [1,2,3], in situ inspection of aero-engines [4,5,6], and space exploration [7]. However, their inherent compliance renders the precise modeling of geometric configurations and dynamic characteristics a formidable challenge. To address this issue, a variety of modeling approaches have been proposed, such as those based on classical beam theory [8,9,10], the absolute node coordinate method (ANCF) [11], and the arbitrary Lagrange–Eulerian (ALE)-ANCF [12,13,14,15,16,17]. Notably, modeling techniques founded on Cosserat rod theory have proven capable of accurately capturing the complex deformations of TDCRs including bending, extension, shear, and torsion—and have gradually emerged as one of the mainstream methodologies in this domain [18].

TDCR models based on Cosserat rod theory typically assume that tendon forces are continuously and uniformly distributed along the rod’s length. This assumption has been successfully applied to a continuum or soft robots in which tendons are embedded within homogeneous media (e.g., silicone) [19]. In modeling a TDCR, methods grounded in Cosserat rod theory generally adopt either a Newtonian framework or a Lagrangian framework [20]. In particular, the Newtonian approach relies on force and moment equilibrium, directly formulating the differential balance equations for slender rods through the Newton–Euler equations. Notably, Rucker et al. [21,22] developed static and dynamic models for TDCRs with arbitrary tendon configurations based on the Cosserat rod framework, systematically deriving the relationship between tendon tension and rod deformation. By numerically solving these differential equations, the pose configurations of a TDCR under various tendon actuation conditions can be accurately determined, thereby providing a robust theoretical foundation for their control and design. In recent years, the Newtonian constant-strain Cosserat rod approach has also been extended to the modeling of rod-actuated continuum robots [23].

The Lagrangian approach, grounded in the energy principle, derives the dynamic model of a TDCR by formulating the system’s Lagrangian density function and applying the variational principle. Renda et al. [24] applied the Cosserat rod model to the dynamic modeling of a TDCR with variable cross-sections by discretizing the continuum in a body-fixed coordinate system, thereby proposing a discrete dynamic modeling method for a TDCR. In subsequent work, a TDCR model based on Cosserat rod theory was developed under the assumption of piecewise constant strain [25]. However, because the actual strain distribution in a TDCR is not constant, Boyer et al. [26] introduced a parameterized strain method to accommodate non-constant strain conditions and further applied the proposed TDCR model to the optimization and analysis of control systems [27]. Extending this line of research, they subsequently applied the Cosserat rod-parameterized strain method to parallel continuum robots [28] and to the modeling of rigid–flexible hybrid structures [29]. In a recent collaborative study, a finite element-like local strain basis was proposed for reduced-order modeling of rigid–flexible hybrid robots [30]. Building on this foundation, Anup et al. [31] further advanced a geometric variable strain method. To further enable the control applications of a TDCR, Li et al. [32] proposed a piecewise linear strain model, which was subsequently applied in control studies [33,34]. Moreover, Xun et al. [35] extended the piecewise linear strain model to investigate contact points during environmental interactions.

In the friction modeling of a TDCR, Yasin et al. [36] relied on external joint force sensing combined with support vector regression to compensate for friction losses. However, their approach depends on experimental data and lacks a physics-based global friction model, making it challenging to capture the dynamic variations and coupling effects of friction accurately. Yuan et al. [37] adopted a piecewise constant curvature assumption for friction modeling, where friction force calculations primarily depend on the tension variations in the drive cables between adjacent segments and a Coulomb friction model is employed to characterize the contact friction between the cables and the guiding channels. Raimondi et al. [38] compared various assumption-based modeling strategies and, by optimizing friction coefficients through experimental validation, investigated the friction and hyperelastic effects in tendon-driven continuum robots. Their study revealed that friction influences the distribution of tendon tension, leading to uneven bending angles across different joints. Liu et al. [39] proposed a real-time dynamic modeling method based on the principle of virtual work and the concentrated displacement model, incorporating friction effects in the tendon-driven system via a Capstan friction model. In contrast to friction models based on the Cosserat rod approach, these methods exhibit a significant drawback: they do not provide a sufficiently global continuous representation of friction forces. This shortcoming impedes the accurate description of TDCR shear effects and the precise consideration of three-dimensional deformations, thereby limiting the computational accuracy and generalizability for structures with complex variable curvatures. Nonetheless, in a recent study, Feliu-Talegon et al. [40] leveraged Cosserat rod theory to propose a dynamic shape estimation algorithm that utilizes tendon length variations and actuator forces in conjunction with a Capstan friction model to estimate the shape of soft robotic arms. However, this research is based on continuous Cosserat rod modeling and does not elaborate on more detailed aspects, such as the directional characteristics of friction forces.

Under identical configurations, a thorough friction analysis is critical for constructing realistic TDCR models, as it necessitates fully accounting for the impact of friction forces on the robot’s configuration. However, current research on a TDCR based on Cosserat rod theory suffers from two significant limitations: first, the disks are assumed to be continuously stacked along the central backbone, thereby neglecting friction effects [20], and second, friction is treated in an overly simplified manner within continuous Cosserat rod models [40]. To address these issues, this paper integrates Cosserat rod theory with classical finite element techniques to improve the modeling of practical problems. Specifically, we develop a tendon actuation model based on discretely distributed forces, abandoning the conventional assumption that driving forces are uniformly and parallelly distributed along the central backbone, and instead propose a TDCR model driven by discrete point forces. Moreover, to tackle the friction between the tendons and the disk guide holes, we introduce a nonlinear friction force analysis to enhance the model’s capacity to describe contact mechanical behavior accurately. Compared with classical TDCR models, simulation and experimental studies demonstrate that the proposed approach exhibits superior effectiveness and accuracy in practical applications.

This paper is organized as follows. In Section 2, the TDCR model is established by first introducing the theoretical framework of the Cosserat rod model, then identifying the scientific questions arising from classical TDCR modeling, subsequently constructing a discrete piecewise linear tendon TDCR model without friction, and finally extending this model to incorporate frictional effects between the tendon and the disks. Section 3 presents numerical simulations and experimental investigations. Numerical simulations were conducted for various tendon routing based on the proposed model, followed by the development of an experimental platform. A comparative analysis of the simulation and experimental results was then performed to verify the model’s effectiveness. Section 4 discusses and compares the simulation and experimental findings obtained under different tendon configurations and identifies the model’s limitations. Finally, Section 5 summarizes the research and proposes directions for future work.

## 2. Modeling

### 2.1. Cosserat Rod Model

Traditional continuum mechanics offers two fundamental frameworks for describing physical systems: the Lagrangian description, which emphasizes material positions, and the Eulerian description, which adopts a spatial perspective. The Cosserat rod theory extends these frameworks by incorporating materials’ microstructural orientation, thereby enhancing continuum mechanics’ modeling capabilities [41]. In recent years, integrating differential geometry with the Cosserat rod model has significantly advanced multibody dynamics, particularly in addressing challenges related to nonlinear large deformations and complex contact mechanics [42]. Building on this foundation, significant progress in TDCR modeling has been made by developing theoretical models based on the Cosserat rod framework from various mechanical and energetic perspectives [21,26]. The classical Cosserat rod formulation provides a mathematical representation of the backbone configuration of a TDCR, which can be written as follows:

(1) $$C=\left\{g\left(s\right)=\left[\begin{array}{cc} R(s) & p(s)\\ 0 & 1 \end{array}\right]\in SE\left(3\right)\right\}.$$

In this formulation (1), C denotes the configuration space of the Cosserat rod, and g(s)∈SE(3) is the configuration matrix of the backbone, with SE(3) being the special Euclidean group. The variable *s* is the arc length parameter of the backbone, $p\left(s\right)\in R^{3}$ represents the position vector, and R(s)∈SO(3) is the rotation matrix. By differentiating the configuration in Equation (1), the differential kinematic formulation is obtained as follows:

(2) $$g^{\prime }\left(s\right)=g\left(s\right)\hat{\xi }\left(s\right)$$

where ${\left(\cdot \right)}^{\prime }$ denotes the derivative with respect to the arc length *s*; $\xi \left(s\right)={\left[K^{⊤}\left(s\right),\Gamma^{⊤}\left(s\right)\right]}^{⊤}\in R^{6}$ represents the generalized strain vector; $\hat{\left(\cdot \right)}$ maps a vector in $R^{6}$ to a 4×4 matrix; $K\left(s\right)\in R^{3}$ is the angular strain; and $\Gamma \left(s\right)\in R^{3}$ is the linear strain. Furthermore, based on the definition in Equation (2), the differential kinematics can be expanded as follows:

(3) $$p^{\prime }\left(s\right)=R\left(s\right)\Gamma \left(s\right),\;R^{\prime }\left(s\right)=R\left(s\right)\tilde{K}\left(s\right)$$

In Equation (3), the operator $\tilde{\left(\cdot \right)}$ denotes the mapping from a 3×1 column vector to its corresponding 3×3 skew-symmetric matrix. For the backbone of the TDCR, under the assumption that inertia is neglected, the equilibrium equations in the local coordinate system are unified and expressed through the following force and moment balance equations:

(4) $$\Lambda_{\mathrm{int}}^{\prime }\left(s\right)={\mathrm{ad}}_{\xi }^{⊤}\Lambda_{\mathrm{int}}\left(s\right)-\bar{F}\left(s\right)$$

In the above equation, the term $\Lambda_{\mathrm{int}}\left(s\right)\in R^{6}$ denotes the distributed generalized internal forces, $\bar{F}\left(s\right)$ represents the total external forces, and ${\mathrm{ad}}_{\xi }$ indicates the adjoint operator in the corresponding Lie algebra. In the local coordinate system, the generalized internal forces $\Lambda_{\mathrm{int}}\left(s\right)$ and the generalized strain ξ(s) satisfy the following constitutive relation:

(5) $$\Lambda_{\mathrm{int}}\left(s\right)=H\left(\xi \left(s\right)-\xi_{0}\right)$$

In this formulation, the Hooke matrix is defined as $H=\mathrm{diag}\left(GJ_{1},\;EI_{1},\;EI_{2},\;EA,\;GA,\;GA\right)$, with *G* the shear modulus, *E* the elastic modulus, and *A* the cross-sectional area. The moments of inertia about the principal axes of the cross-section are denoted by $I_{1}$ and $I_{2}$, and the polar moment of inertia is given by $J_{1}=I_{1}+I_{2}$.

Following the Ritz method, the generalized strain in Equation (5) can be represented as follows [43]:

(6) $$\xi \left(s\right)=\xi_{0}+\Phi \left(s\right)q$$

Within this formulation, $\xi_{0}$ represents the generalized strain in the reference state, Φ(s) denotes the shape functions, and q refers to the discretized nodal generalized coordinates. Additionally, the boundary conditions for Equation (4) are given as follows:

(7) $$g\left(0\right)=I_{4\times 4},\;\Lambda \left(L^{k}\right)=F_{+},\;k=1,2,\ldots ,N-1$$

where *k* denotes the index of the discretized continuum segment, *N* represents the number of disks, g(0) is the initial pose matrix at the starting point of the arc length parameter (s=0), and $I_{4\times 4}$ is the 4×4 identity matrix. $F_{+}$ represents the generalized external force at the end of a continuous arc segment, $s=L^{k}$ corresponds to the *k*-th continuum segment, and *L* is the total length of the TDCR backbone.

### 2.2. Methodological Contributions

Equation (4) represents the classical Cosserat rod model, which also serves as the foundational model for the TDCR backbone. Various numerical solutions and parameterization strategies have been developed to construct and solve the TDCR model from the backbone model. Among them, two commonly used approaches are the Newton–Euler iterative numerical method [21] and the variational solution strategy based on the Lagrangian formulation [27]. The choice and application of these methods typically depend on specific requirements, computational cost, and desired numerical stability. A comparative study in [20] evaluated these two methods’ modeling and solution performance for TDCR systems, highlighting their respective advantages, limitations, and applicability. However, regardless of the solution strategy employed, within the classical modeling framework of the Cosserat rod-based TDCR model, the system can ultimately be reduced to a state equation resembling the classical Cosserat rod formulation, which can be expressed as the following unified equilibrium equation:

(8) $$\Lambda_{\mathrm{int}}^{\prime }\left(s\right)={\mathrm{ad}}_{\xi }^{⊤}\Lambda_{\mathrm{int}}\left(s\right)-\left(\Lambda_{e}\left(s\right)+\Lambda_{a}\left(s\right)\right)$$

where $\Lambda_{\mathrm{int}}\left(s\right)$ represents the internal forces and moments distributed along the rod parameter *s*, $\Lambda_{e}\left(s\right)$ denotes the external wrench acting on the backbone, and $\Lambda_{a}\left(s\right)$ corresponds to the tendon-driven forces and the resulting distributed torques.

**Figure 1:**
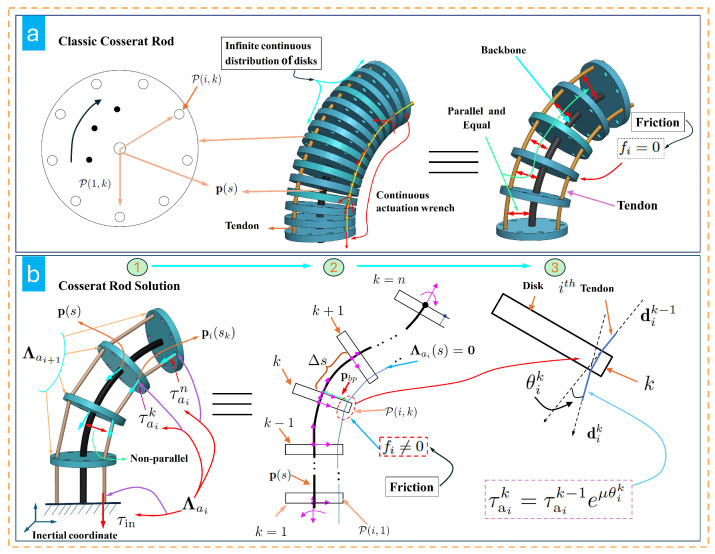
Schematics of traditional TDCR and our proposed TDCR configuration.

In classical Cosserat rod-based TDCR modeling, two main assumptions are typically made: (i) the tendon driving force $\Lambda_{a}$ is treated as a continuous function defined along the entire length of the tendon, and the tendon is assumed to be geometrically parallel to the backbone, as shown in Figure 1a; and (ii) friction between the tendon and the disk holes is neglected. However, practical TDCR systems deviate from these assumptions. Tendons follow piecewise linear segments under tension and are not parallel to the curved backbone. Furthermore, no external driving force exists other than the tendon–disk hole contact, and the frictional effects between the tendon and the holes cannot be ignored (Figure 1b). These discrepancies highlight the limitations of the classical TDCR model in real-world applications.

To tackle these issues, the frictional interactions between the tendon and the disk holes were thoroughly incorporated, leading to the following enhancements: (1) The tendon driving force $\Lambda_{a}\left(s\right)$ is defined discretely, with straight line segments between adjacent disks. (2) The model no longer assumes that the tendon driving force is parallel to the backbone; instead, a discrete concentrated force actuation scheme is employed. (3) Frictional forces between the tendons and the disk holes are incorporated to capture the system’s nonlinear behavior. (4) Apart from this tendon–disk friction, no other external driving factors are assumed, aligning more closely with the actual physical system. (5) Comparisons of the simulation and experimental results with those from the classical TDCR model are conducted to verify the effectiveness and accuracy of the improved model.

### 2.3. Discrete Tendon TDCR Model Without Friction

The TDCR is a robotic system that achieves motion through the actuation of a continuum backbone by tendons arranged in various configurations. Classical TDCR models assume that disks are continuously distributed along the backbone, and the backbone deformation is driven by tendons. In traditional TDCR models, tendons are simplified into a continuous distribution model that establishes a derivative relationship with the backbone, assuming that the driving force exerted by the tendons is present at any position. The tendon driving force wrench expression of the *i*-th tendon in the local coordinate system is given as follows [21]:

(9) $$\Lambda_{a_{i}}={\left[\begin{array}{c} {\left[\begin{array}{c} -\left(P_{i}\left(s\right)-p\left(s\right)\right)\times \left(-\tau_{a_{i}}\frac{{\hat{P}}_{i}^{\prime }{\left(s\right)}^{2}P_{i}^{\prime \prime }\left(s\right)}{∥P_{i}^{\prime }{\left(s\right)∥}^{3}}\right) \end{array}\right]}^{⊤}\\ {\left[\begin{array}{c} -\tau_{a_{i}}\frac{{\hat{P}}_{i}^{\prime }{\left(s\right)}^{2}P_{i}^{\prime \prime }\left(s\right)}{∥P_{i}^{\prime }{\left(s\right)∥}^{3}} \end{array}\right]}^{⊤} \end{array}\right]}^{⊤}$$

where $\Lambda_{a_{i}}$ represents the driving wrench vector when the tendon contacts the end disk or the intermediate distributed holes. ${\left(\cdot \right)}^{\prime \prime }$ denotes the second derivative with respect to the arc length *s*. $P_{i}\left(s\right)$ is the position vector of the *i*-th tendon, and $\tau_{a_{i}}$ indicates the magnitude of the driving force exerted by the *i*-th tendon.

It was assumed in Equation (9) that the tendon driving wrench function is continuous and the friction is negligible. Under these assumptions, the tendon driving wrench $\Lambda_{a_{i}}$ remains uniformly continuous at any position along the tendon, and the tension is identical throughout the tendon length, as illustrated in Figure 1a. However, in practical applications, disks are typically installed on the backbone at either equal or unequal intervals, causing the tendon to form piecewise linear segments between adjacent disks. The distance of these segments from the backbone is not constant. To address this discrepancy and better approximate the actual physical model, a series of studies was conducted in this work.

In constructing the model, the following assumptions were required: (1) The tendon holes on each disk are discretely distributed along the backbone according to the arc length parameter *s*, where k=1,⋯,N denotes the number of spaced disks; (2) each tendon passes through the tendon holes and forms piecewise linear segments between adjacent disks; (3) the tendon is regarded as inextensible, and any elongation due to tension is neglected; (4) the influence of disk thickness is disregarded; and (5) the tendon is always under tension in the TDCR. For accurate modeling of the tendon direction at each disk, the unit direction vector of the *i*-th tendon’s driving force at the *k*-th disk is given by the following:

(10) $$d_{i}^{k}=\frac{P(i,k-1)-P(i,k)}{∥P(i,k-1)-P(i,k)∥}$$

When the friction between tendons and the disk tendon holes is negligible, the frictional forces at discrete points along each tendon and spacer disk are uniformly distributed. For instance, the magnitudes of the adjacent driving forces at the (k−1)-th and (k+1)-th disks corresponding to the *i*-th tendon can be expressed as follows:

(11) $$\tau_{a_{i}}^{k-1}=\tau_{a_{i}}^{k}=\tau_{a_{i}}^{k+1}$$

The TDCR is driven by discrete concentrated forces applied at the disks. These driving concentrated forces, relative to the backbone of the TDCR, constitute a driving wrench that includes discrete concentrated force and point moments. Significantly, the driving wrench of the TDCR varies across different driving points. For example, the driving wrenches at the two ends and the middle section of the TDCR are not identical due to differing boundary conditions. Consequently, incorporating the continuous driving wrench Equation (9), for the case of discrete concentrated force actuation, the driving wrench at the *k*-th disk along the *i*-th tendon, excluding the starting end of the backbone and the base disk, can be expressed as follows:

(12) $$\Lambda_{a_{i}}^{k}={\left[\begin{array}{c} {\left[\begin{array}{c} \left(P\left(i,k\right)-p\left(s^{k}\right)\right)\times \tau_{a_{i}}^{k}d_{i}^{k} \end{array}\right]}^{⊤}\\ {\left[\begin{array}{c} \tau_{a_{i}}^{k}d_{i}^{k} \end{array}\right]}^{⊤} \end{array}\right]}^{⊤}$$

where $\tau_{a_{i}}^{k}$ denotes the magnitude of the tendon force at the tendon hole of the *k*-th disk along the *i*-th tendon in the local frame. Furthermore, based on Figure 1b, the discrete expression for the driving force of the *i*-th tendon in practical TDCR applications is as follows:

(13) $$\Lambda_{a_{i}}=\Lambda_{a_{i}}^{0}\delta \left(s\right)+\sum_{k=1}^{n-1}\Lambda_{a_{i}}^{k}\delta \left(s-k\Delta s\right)+\Lambda_{a_{i}}^{n}\delta \left(s-L\right)$$

where δ is the impulse function that defines the discrete driving forces acting on different disk points. Each disk is represented by the function δ(s), which specifies the location and distribution characteristics of the driving forces. The magnitude of the discrete force at the intermediate disks is denoted as $\tau_{a_{i}}^{k}$, corresponding to the discrete point s=kΔs, where *k* is the index of the discrete point and Δs is the step size. Additionally, $\tau_{a_{i}}^{0}$ and $\tau_{a_{i}}^{n}$ represent the tendon driving forces at the starting position s=0 and the end position s=L, respectively, with their distributions described by $\delta (s-s_{0})$ and δ(s−L). The expression for the tendon driving wrench $\Lambda_{a_{i}}$ at the discrete points of each disk is given by the following:

(14) $$\Lambda_{a_{i}}\left(s\right)=\left\{\begin{array}{cc} {\left[{\left(\left(P\left(i,k\right)-p\left(s^{k}\right)\right)\times \tau_{\mathrm{in}}d_{0}\right)}^{⊤};{\left(\tau_{\mathrm{in}}d_{0}\right)}^{⊤}\right]}^{⊤}, & s=0,\\ P\left(i,k\right)-p\left(s^{k}\right))\times \tau_{a_{i}}^{k}d_{i}^{k}{{)}^{⊤};{\left(\tau_{a_{i}}^{k}d_{i}^{k}\right)}^{⊤}]}^{⊤}, & s=k\Delta s,\;k=1,2,\ldots ,n-1,\\ P\left(i,k\right)-p\left(s^{k}\right))\times \tau_{a_{i}}^{n}d_{i}^{n}{{)}^{⊤};{\left(\tau_{a_{i}}^{n}d_{i}^{n}\right)}^{⊤}]}^{⊤}, & s=L,\\ 0, & \mathrm{Other}\;\mathrm{position} \end{array}\right.$$

In the above formula, $\tau_{\mathrm{in}}$ represents the initial pulling force applied by the motor to the tendon, typically acting at the starting position s=0. $d_{0}={\left[0,0,1\right]}^{⊤}$ is the directional vector of the initial pulling force. $\tau_{a_{i}}^{k}$ denotes the magnitude of the force applied at the discrete disk point s=kΔs, which is uniformly distributed at all discrete points s=kΔs under the assumption of negligible friction. $\tau_{a_{i}}^{n}$ refers to the force or tension applied at the system’s endpoint s=L, typically exerted by terminal equipment or an external load.

### 2.4. Discrete TDCR Model Considering Friction

In a TDCR, tendons driven by motors create the bending configuration through a combination of tension, retraction, and frictional interactions with the disk holes. Under quasi-static conditions, even when the TDCR maintains the same bending configuration, the direction of the frictional forces between the tendons and the disk holes may vary. For instance, as tendons pass through a series of disk holes along a curved path, tightening the tendons to induce bending causes frictional forces to be exerted on the disks, with the disks generating reaction forces in the opposite direction. Conversely, during tendon retraction, the tendon tension aligns with the direction of the frictional force. In a quasi-static model with a known TDCR configuration, the frictional forces between tendons and disk holes may act in the same or opposite directions. Therefore, accurately determining and calculating the direction of frictional forces is critical for the precise modeling and analysis of the mechanical behavior of TDCR.

Differing from conventional TDCR modeling, the proposed model discretizes the tendon into n=N−1 linear segments, where *N* represents the number of disks. The tendon length is expressed as the sum of the lengths of these segments. Although the model considers quasi-static conditions, it is valid to define the shape of the *i*-th tendon at time *t* using the parameter *s* in the discretized quasi-static scenario. Assuming a quasi-static condition as t→0, the distance between the *k*-th and (k−1)-th disks along the *i*-th tendon can be geometrically defined as follows:

(15) $$∥D_{i}^{k}\left(t\right)∥=∥P\left(i,k,t\right)-P\left(i,k-1,t\right)∥$$

To determine the rate of change in the total tendon length, directly using finite difference summation to assess the sign of ${\dot{l}}_{i}\left(t\right)$ is challenging. This difficulty arises because velocity differences encompass components in multiple directions, while only the component along the segment path contributes to actual length variation. Components perpendicular to the segment path merely alter the shape without affecting the path length. However, during the motion of a TDCR, the tendons are typically under tension. Specifically, when ${\dot{l}}_{i}\left(t\right)>0$, the tendon is in a releasing state, and the friction force generated by the guide hole on the disk opposes this releasing tendency, aligning in the same direction as the tensile force acting on the tendon. Conversely, when ${\dot{l}}_{i}\left(t\right)<0$, the tendon is in a tightening state, and the friction force exerted by the guide hole resists further contraction of the tendon, resulting in a friction force direction opposite to that of the tensile force. Therefore, it is necessary to compute the derivative of the total length of the *i*-th tendon:

(16) $${\dot{l}}_{i}\left(t\right)=\sum_{k=1}^{n}\frac{{\left(D_{i}^{k}\left(t\right)\right)}^{⊤}\cdot \left(\dot{P}\left(i,k,t\right)-\dot{P}\left(i,k-1,t\right)\right)}{∥D_{i}^{k}\left(t\right)∥}$$

By determining the sign of ${\dot{l}}_{i}\left(t\right)$, the direction of the friction force can be identified, which is crucial for developing a TDCR model that closely aligns with real-world conditions. To determine the sign of ${\dot{l}}_{i}\left(t\right)$, the total length of the *i*-th tendon must be constructed based on the kinematics of the Cosserat rod. According to the literature [28], the kinematics of the *i*-th tendon can be derived using the classical relationship $\eta^{\prime }=-{\mathrm{ad}}_{\xi }\eta +\dot{\xi }$ and ${\mathrm{ad}}_{\xi }={\mathrm{Ad}}_{g^{-1}}{\mathrm{Ad}}_{g}^{\prime }$, combined with the strain–stress relationship expressed in Equation (6). The resulting kinematic expression for the *i*-th tendon is as follows [44]:

(17) $$\eta_{i}\left(s\right)={\mathrm{Ad}}_{g_{i}^{-1}\left(s\right)}{\mathrm{Ad}}_{g_{i}0}\eta_{i0}+{\mathrm{Ad}}_{g_{i}^{-1}\left(s\right)}\int_{0}^{s}{\mathrm{Ad}}_{g_{i}\left(x\right)}\Phi_{i}\;dX\;{\dot{q}}_{i}$$

Regarding Equation (17), further details are provided in [26]. Additionally, based on the relationship ${\dot{g}}_{i}=g_{i}\eta_{i}$ in the Cosserat rod model, where $\eta_{i}=\left[\omega_{i},v_{i}\right]\in R^{6}$ represents the velocity vector of the *i*-th tendon, the decomposition of $\eta_{i}\left(s\right)$ is performed to derive $v_{i}\left(s\right)$. Consequently, Equation (17) can be reformulated as follows:

(18) $$\eta_{i}\left(s\right)\;=\;\eta_{i,\mathrm{const}}\left(s\right)\;+\;\eta_{i,\mathrm{flex}}\left(s\right)={\left[\omega_{i,\mathrm{const}}^{⊤}\left(s\right),v_{i,\mathrm{const}}^{⊤}\left(s\right)\right]}^{⊤}+{\left[\omega_{i,\mathrm{flex}}^{⊤}\left(s\right),v_{i,\mathrm{flex}}^{⊤}\left(s\right)\right]}^{⊤}$$

where, within this formulation, $\eta_{i,\mathrm{const}}\left(s\right)={\mathrm{Ad}}_{\;g_{i}^{-1}\left(s\right)}\;{\mathrm{Ad}}_{\;g_{i0}}\;\eta_{i0}$ represents the velocity of the *i*-th segment relative to the base coordinate system in the reference configuration. The velocity induced by flexible deformation, $\eta_{i,\mathrm{flex}}\left(s\right)$, is expressed as a shape function describing tendon deformation, with ${\dot{q}}_{i}$ denoting the generalized velocity of the flexible coordinates. The total velocity of the *i*-th segment at arc coordinate *s* is the sum of the reference velocity and the velocity due to flexible deformation, given by the following expression:

(19) $$v_{i}\left(s\right)\;=\;v_{i,\mathrm{const}}\left(s\right)\;+\;v_{i,\mathrm{flex}}\left(s\right)$$

Next, $v_{i,\mathrm{const}}\left(s\right)$ is isolated. For $\eta_{i,\mathrm{const}}\left(s\right)$, the following expression is obtained:

(20) $$\eta_{i,\mathrm{const}}\left(s\right)\;=\;{\mathrm{Ad}}_{\;g_{i}^{-1}\left(s\right)}\;{\mathrm{Ad}}_{\;g_{i0}}\;\eta_{i0}={\mathrm{Ad}}_{\;g_{i}^{-1}\left(s\right)}\left[\begin{array}{c} \omega_{0}\\ v_{0} \end{array}\right]$$

Expanding Equation (20) yields the following expression for linear velocity:

(21) $$v_{i,\mathrm{const}}\left(s\right)\;=\;-\;\tilde{P}\left(i,k\right)\left(s\right)\;R_{i}^{-1}\left(s\right)\;\omega_{0}\;+\;R_{i}^{-1}\left(s\right)\;v_{0}$$

Further analysis of $\eta_{i,\mathrm{flex}}\left(s\right)$ yields the following:

(22) $$\eta_{i,\mathrm{flex}}\left(s\right)={\mathrm{Ad}}_{\;g_{i}^{-1}\left(s\right)}\int_{0}^{s}{\mathrm{Ad}}_{g(X)}\Phi_{i}\left(X\right)\;dX\;{\dot{q}}_{i}\left(t\right)$$

However, in practical numerical discretization, the Φ-dimensional parameters can be sparsely configured to meet the requirements of dimensionality reduction for various applications. For example, it can be reduced to a single degree of freedom, considering only tendon elongation. By incorporating different Φ functions and combining them with Equation (22), the expression for the linear velocity $v_{i,\mathrm{flex}}$ is derived as follows:

(23) $$v_{i,\mathrm{flex}}\left(s\right)={\mathrm{Ad}}_{\;g_{i}^{-1}\left(s\right)}\int_{0}^{s}{\mathrm{Ad}}_{g(X)}\Phi_{i}\left(X\right)\;dX\;{\dot{q}}_{i}\left(t\right)$$

Therefore, combining Equations (21) and (23), Equation (19) is updated as follows:

(24) $$v_{i}\left(s\right)=v_{i,\mathrm{const}}\left(s\right)+{\mathrm{Ad}}_{\;g_{i}^{-1}\left(s\right)}\int_{0}^{s}{\mathrm{Ad}}_{g(X)}\Phi_{i}\left(X\right)\;dX$$

Subsequently, the relationship in Equation (24) is substituted into Equation (16), and the simplified expression is updated as follows:

(25) $${\dot{l}}_{i}\left(t\right)=C\;+\;\sum_{k=1}^{n}\left(J_{i}^{k}\left(t,s\right)\;{\dot{q}}_{i}^{k}-J_{i}^{k-1}\left(t,s\right)\;{\dot{q}}_{i}^{k-1}\right)$$

where

$$C=\sum_{k=1}^{n}\left[u_{k}{\left(t\right)}^{⊤}\;R_{i}^{k}\;v_{i,\mathrm{const}}^{k}\left(s\right)-u_{k}{\left(t\right)}^{⊤}\;R_{i}^{k-1}\;v_{i,\mathrm{const}}^{k-1}\left(s\right)\right]$$

**Figure 2:**
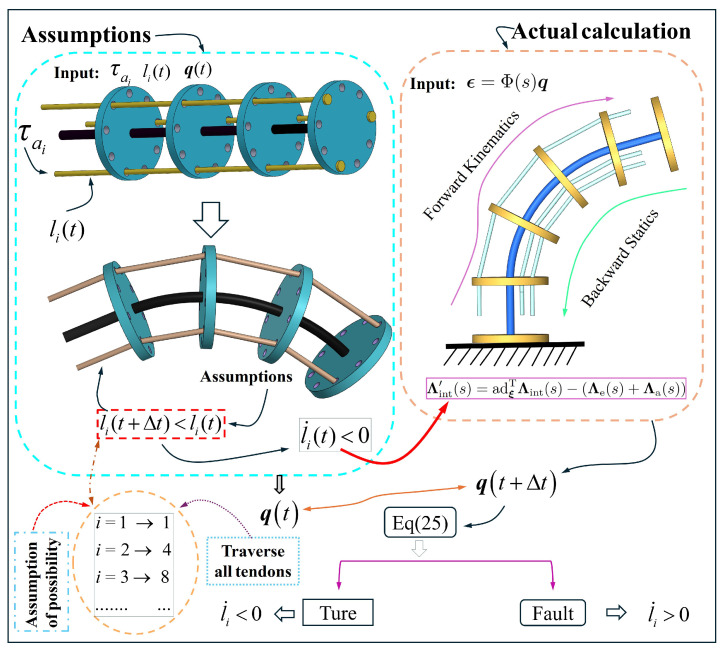
Algorithm for determining tendon contraction or retraction.

However, for the currently derived equation, the sign of ${\dot{l}}_{i}$ cannot be directly determined. To address this, an algorithm is implemented to perform the relevant computations, with the process illustrated in Figure 2. At the initial instant of time *t*, the total length of the tendon $l{\left(i\right)}_{t}$ and the corresponding generalized coordinates q are known. Under the influence of the tendon driving force $\tau_{a_{i}}$, the system transitions to a new configuration. At this stage, it is necessary to iterate through all the possible tendon length combinations for the TDCR at an instant of time t+Δt. In each iteration, ${\dot{l}}_{i}<0$ is assumed, and this assumption is substituted into the TDCR system model to calculate the new generalized coordinates q(t+Δt). Subsequently, by comparing the values of Δq between an instant of time *t* and t+Δt, and incorporating Equation (25), the correctness of the assumption is evaluated. This process determines the sign of ${\dot{l}}_{i}$ for each tendon.

During the motion of the TDCR, in the absence of friction, it is generally assumed that the tendon is only subjected to tensile forces, with its direction consistently aligned along the tendon. However, when considering the friction between the tendon and the disk guide hole, the direction of the friction force may depend on whether the tendon is tightening or releasing. When the tendon tightens, the friction between the tendon and the disk guide hole resists the tightening motion. At this point, the unit directional vector of the friction force between the tendon and the disk holes at the *k*-th and (k−1)-th disks is given by the following:

(26) $$d_{i-}^{k}=\frac{P\left(i,k\right)\left(s^{k}\right)-P\left(i,k\right)\left(s^{k-1}\right)}{∥P\left(i,k\right)\left(s^{k}\right)-P\left(i,k\right)\left(s^{k-1}\right)∥},\;d_{i-}^{k+1}=\frac{P\left(i,k\right)\left(s^{k+1}\right)-P\left(i,k\right)\left(s^{k}\right)}{∥P\left(i,k\right)\left(s^{k+1}\right)-P\left(i,k\right)\left(s^{k}\right)∥}$$

When the tendon is released, the friction between the tendon and the disk resists the release motion. In this case, the direction of the friction force aligns with the tensile force acting on the tendon. The unit directional vector of the friction force between the tendon and the disk holes at the *k*-th and (k−1)-th disks is given by the following:

(27) $$d_{i+}^{k}=\frac{P\left(i,k\right)\left(s^{k-1}\right)-P\left(i,k\right)\left(s^{k}\right)}{∥P\left(i,k\right)\left(s^{k-1}\right)-P\left(i,k\right)\left(s^{k}\right)∥},\;d_{i+}^{k+1}=\frac{P\left(i,k\right)\left(s^{k}\right)-P\left(i,k\right)\left(s^{k+1}\right)}{∥P\left(i,k\right)\left(s^{k}\right)-P\left(i,k\right)\left(s^{k+1}\right)∥}$$

When assuming that the disk is sufficiently thin and the edges of the holes are smooth, the friction behavior between the tendon and the walls of the disk holes conforms to the quasi-static model characteristics of a flexible body on a curved surface. Based on the theoretical assumptions of the Capstan equation regarding the frictional force distribution of a flexible body along a curved surface, it is reasonable to employ the Capstan equation for friction modeling in this study. The consistent friction coefficient of the tendon and the disk’s hole material is denoted as μ. The mass and elastic effects of the tendon are neglected, and it is assumed that the tendon is in a critical state of impending motion, representing a quasi-static condition where $f_{s}=\mu_{s}N$. Using an approximation derived from the Capstan equation, the expression for driving the tendon through the *k*-th hole in the disk is given as follows [45]:

(28) $$\tau_{a_{i}}^{k}=\tau_{a_{i}}^{k-1}e^{\mu \theta_{i}^{k}}$$

where $\tau_{a_{i}}^{k-1}$ represents the driving force magnitude of the *i*-th tendon at the (k−1)-th disk, and $\tau_{a_{i}}^{k}$ represents the driving force magnitude of the *i*-th tendon at the *k*-th disk. $\theta_{i}^{k}$ denotes the angle between adjacent tendons and the hole of the disk. Based on Equations (26) and (27), the direction of the friction force between the tendon and the hole varies depending on the sign of the tendon variation rate in the actuator section. Therefore, when the total length of the *i*-th tendon is either greater than or less than zero, the angular relationship between adjacent tendons at the corresponding *k*-th disk is as follows:

(29) $$\theta_{i}^{k}=\left\{\begin{array}{cc} \theta_{i-}^{k}=\arccos\left(d_{i-}^{k+1}\cdot d_{i-}^{k}\right), & {\dot{l}}_{i}<0,\\ \theta_{i+}^{k}=\arccos\left(d_{i+}^{k+1}\cdot d_{i+}^{k}\right) & {\dot{l}}_{i}>0 \end{array}\right.$$

Considering the direction of the relative motion friction force between the tendon and the disk hole in practical applications, Equation (28) is further updated as follows:

(30) $$\tau_{a_{i}}^{k}=\tau_{a_{i}}^{k-1}\exp\left(\mathrm{sgn}\left({\dot{l}}_{i}\right)\cdot \mu \theta_{i}^{k}\right)$$

where $\tau_{a_{i}}^{k}$ represents the driving force between the *k*-th disk hole and the tendon, while $\tau_{a_{i}}^{k-1}$ denotes the frictional force between the (k−1)-th disk hole and the tendon. The term $\mathrm{sgn}({\dot{l}}_{i})$ controls the direction of the frictional force, corresponding to the relative motion direction of the contacting objects. It determines the frictional force direction based on changes in the rope length or the direction of the tendon tension force. When ${\dot{l}}_{i}>0$, the frictional force aligns with the direction of the tendon’s driving tension force, and the sign function has a value of 1. Conversely, when ${\dot{l}}_{i}<0$, the frictional force opposes the direction of the tendon’s driving tension force, and the sign function has a value of −1.

Considering the friction between the tendon and the disk holes, the tendon’s driving force experiences attenuation as it passes through each hole, as described by Equation (28). With an initial tension of $\tau_{a_{i}}^{0}$, starting from the first disk and proceeding sequentially, the expression for the driving force of the *i*-th tendon after passing through n−1 disks is given as follows:

(31) $$\tau_{a_{i}}^{n}=\tau_{a_{i}}^{0}\exp\left(\sum_{k=1}^{n-1}\mathrm{sgn}\left({\dot{l}}_{i}\right)\mu \theta_{i}^{k}\right)$$

In conjunction with Equation (26), the wrench of the driving force between the tendon and the hole at the *k*-th disk can be expressed as follows, provided that the tendon undergoes continuous shortening:

(32) $$\Lambda_{a_{i}}^{k}={\left[\begin{array}{c} {\left[\begin{array}{c} \left(P\left(i,k\right)-p\left(s^{k}\right)\right)\times \tau_{a_{i}}^{k}d_{i-}^{k} \end{array}\right]}^{⊤}\\ {\left[\begin{array}{c} \tau_{a_{i}}^{k}d_{i-}^{k} \end{array}\right]}^{⊤} \end{array}\right]}^{⊤}$$

In this equation, $\Lambda_{a_{i}}^{k}$ represents the wrench on the *i*-th tendon at the *k*-th disk. The term $\tau_{a_{i}}^{k}d_{i-}^{k}$ represents the driving force vector between the tendon and the tendon hole at the *k*-th disk, where the magnitude is determined by the driving force $\tau_{a_{i}}^{k}$, and $d_{i-}^{k}$ is the unit vector of the frictional force at the hole of the *i*-th tendon on the *k*-th disk during tendon contraction.

Based on Equation (32), the discrete form of the concentrated force driving between the tendon and the disk hole is expressed as follows:

(33) $$\Lambda_{a_{i}}=\Lambda_{a_{i}}^{0}\delta \left(s\right)+\sum_{k=1}^{n-1}\Lambda_{a_{i}}^{k}\delta \left(s-k\Delta s\right)+\Lambda_{a_{i}}^{n}\delta \left(s-L\right)$$

The boundary conditions for Equation (33) can be represented as a piecewise function, expressed as follows:

(34) $$\Lambda_{a_{i}}\left(s\right)=\left\{\begin{array}{cc} {\left[{\left(\left(P\left(i,k\right)-p\left(s^{k}\right)\right)\times \tau_{\mathrm{in}}d_{0}\right)}^{⊤};{\left(\tau_{\mathrm{in}}d_{0}\right)}^{⊤}\right]}^{⊤}, & s=0,\\ P\left(i,k\right)-p\left(s^{k}\right))\times \tau_{a_{i}}^{k}d_{i-}^{k}{{)}^{⊤};{\left(\tau_{a_{i}}^{k}d_{i-}^{k}\right)}^{⊤}]}^{⊤}, & s=k\Delta s,\;k=1,2,\ldots ,n-1,\\ P\left(i,k\right)-p\left(s^{k}\right))\times \tau_{a_{i}}^{n}d_{i}^{n}{{)}^{⊤};{\left(\tau_{a_{i}}^{n}d_{i}^{n}\right)}^{⊤}]}^{⊤}, & s=L,\\ 0, & \mathrm{Other}\;\mathrm{position} \end{array}\right.$$

herein, $s_{1},s_{2},\cdots ,s_{n-1}$ represent the boundaries of the segmented intervals. The frictional force $\tau_{a_{i}}\left(s\right)$ of the tendon passing through each disk hole differs in magnitude and direction, which depend on $\mathrm{sgn}({\dot{l}}_{i})$ and the parameter $\mu \theta_{i}^{k}$. Subsequently, when considering the friction between the tendon and the disk holes, and combining Equations (8), (13), and (33), the frictional TDCR model expression is further updated as follows:

(35) $$\Lambda_{\mathrm{int}}^{\prime }\left(s\right)={\mathrm{ad}}_{\xi }^{⊤}\Lambda_{\mathrm{int}}\left(s\right)-\left(\Lambda_{e}\left(s\right)+\Lambda_{a}\left(s\right)\right)$$

with boundary conditions:

$$g\left(0\right)=I_{4\times 4},\;\Lambda \left(L^{k}\right)=F_{+},\;k=1,2,\ldots ,n$$

## 3. Numerical Simulation and Experiment

### 3.1. Numerical Simulation

The numerical simulation algorithm used in this study is based on the method described in the literature [26] and was implemented on a system manufactured by MECHREVO (Shanghai, China), equipped with an Intel i9-13900 processor, 64 GB of RAM, and an RTX 4080 GPU, utilizing MATLAB 2024a for the simulation and analysis. Building upon this algorithm, high-precision simulations of the TDCR under concentrated loads and various tendon-routing configurations were implemented. First, the numerical simulation incorporates friction modeling and supports multiple tendon-routing schemes, including parallel, helical, and convergent configurations. The corresponding tendon functions follow the guidelines presented in [20]. The concentrated load simulation feature was added to the friction model, allowing for a flexible load position and orientation definition to capture complex loading scenarios.

In the TDCR examined here, the backbone was discretized into multiple disks, and three tendons induced deformation through end fixation or friction between the tendons and the disks. The material and geometric parameters were specified during the numerical simulations. For instance, the axial and bending stiffness of the TDCR backbone were governed by Young’s modulus ($E=85\times 10^{9}\;\mathrm{Pa}$) and Poisson’s ratio (ν=0.3), and the shear modulus was given by $G_{j}=\frac{E}{2(1+\nu )}$. The equivalent mass density was $\rho =5600\;{\mathrm{kg}/m}^{3}$, and a friction coefficient of 0.3 was assumed under quasi-static conditions. In terms of geometry, the backbone radius $R_{b}=0.4\times 10^{-3}\;m$ determined the cross-sectional moment of inertia and its stiffness characteristics, affecting both bending capacity and load-bearing capability. The radial distance of each tendon routing and the backbone radius influenced how tendon tension translated into bending moments. By specifying these material parameters and applying a quasi-static framework along with boundary conditions (e.g., Equation (35)), the TDCR was numerically solved. To comprehensively compare the advantages of the proposed model against traditional methods, three simulation modes were investigated: (i) the conventional continuous tendon force model was used as a baseline; (ii) the discrete concentrated force model was adopted for the tendons; and (iii) the discrete concentrated force model was further enhanced by including friction effects.

**Figure 3:**
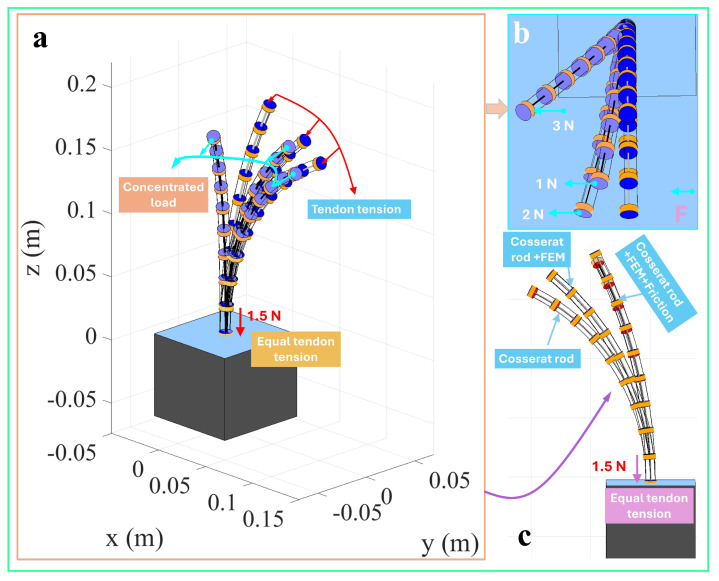
One-segment TDCR driving with concentrated load.

Figure 3 illustrates the three-dimensional configuration of a single-segment TDCR under the combined action of a 1.5 N tendon force and an end-concentrated load. The results predicted by three different modeling approaches are compared: the classical Cosserat rod-based TDCR model, the discrete concentrated force-driven Cosserat rod model, and the discrete concentrated force-driven model incorporating friction effects. Figure 3a presents an isometric view, providing an intuitive representation of the overall deformation under different combinations of concentrated loads and tendon forces. Figure 3b,c highlight the differences in the TDCR configuration from top and side perspectives, emphasizing variations in the planar and vertical directions. The simulation results indicate that the classical Cosserat rod model predicts the most considerable deformation magnitude under the same tendon force. Introducing discrete concentrated force slightly reduces the overall deformation of the TDCR configuration, and further reduction is observed when friction effects are incorporated.

**Figure 4:**
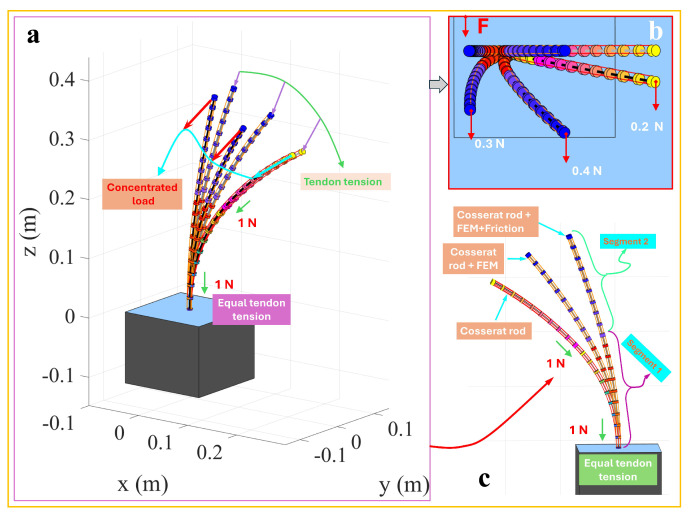
Two-segment TDCR driving with concentrated load.

In complex application scenarios, a TDCR often requires multi-segment structures to apply concentrated loads at different nodes and perform more flexible operations. To address this, the single-segment TDCR was extended to a two-segment structure for simulation. Each segment had a length of 200 mm, resulting in a total length of 400mm. Three tendons were arranged per segment with equal angular spacing and an offset distance of 4.5mm, while the other material parameters remained consistent with the single-segment model. This setup reflected the complexity of multi-segment structures while maintaining parameter comparability. Figure 4a shows the overall deformation of the two-segment TDCR under the combined action of 1 N tendon tension and an external concentrated load. The green arrows indicate the deformation path driven solely by tendon forces, while the cyan arrows depict the additional deformation resulting from the applied concentrated load. Figure 4b presents a close-up top view, illustrating that, as the concentrated load increases from 0.2N to 0.4N, the overall deformation also increases, indicating higher sensitivity of the multi-segment structure to coupled deformations at the end and along its segments. As shown in Figure 4c, comparing the classical Cosserat rod model, the discrete concentrated force-driven model, and the discrete concentrated force-driven model incorporating friction reveals consistent trends. Under identical tendon forces, the classical Cosserat rod model predicted the most considerable deformation magnitude, while the discrete concentrated force-driven model showed reduced deformation. When friction effects were included, the deformation further decreased. These results demonstrate consistent deformation patterns between multi- and single-segment TDCRs under external concentrated loads.

**Figure 5:**
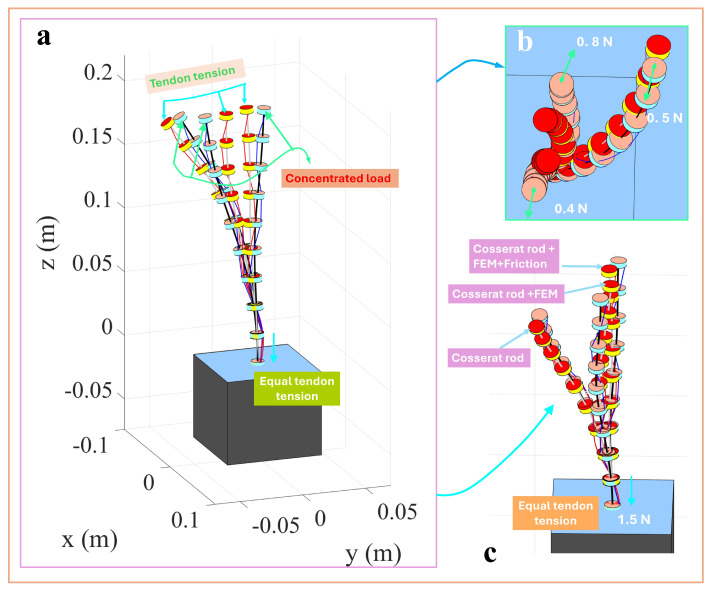
Helical tendon-routing TDCR drive under concentrated load.

This study proposes a helical tendon arrangement to achieve complex spatial deformation without increasing the number of actuators. A single tendon was wound around the backbone in a 720° helical path, while other structural parameters remained consistent with the single-segment model. This configuration reduces the number of independently driven tendons while maintaining strong deformation capabilities. In the simulation, a tendon force of 1.2N was applied to a single helical tendon routing, along with a concentrated load along the *Y*-axis ranging from 0.4N to 0.8N, to investigate deformation characteristics under the helical path. Figure 5a illustrates the overall three-dimensional configuration, while Figure 5b provides an enlarged top view, showing that deformation in the helical segment intensifies as the external load increases. Figure 5c compares the deformation results predicted by three modeling approaches (classical Cosserat rod, discrete concentrated force, and friction-inclusive discrete concentrated force models). Compared to the conclusions drawn for the parallel tendon arrangement, the friction-inclusive discrete concentrated force model predicts minor deformation, the discrete concentrated force model shows slightly larger deformation, and the classical Cosserat rod model predicts the largest deformation amplitude.

TDCRs offer significant advantages in minimally invasive surgery due to their compliance, particularly when convergent tendon routing is used to reduce the size of end effectors, meeting the demands of confined surgical spaces. Based on the material and structural parameters of a single-segment model, a convergent tendon routing was designed in this study. The initial tendon offset was set at 4.5mm, which gradually reduced to 0.15mm at the distal end. The simulations involved a three-step loading sequence: an *X*-axis terminal load of 1.1N, a tendon force of 0.3N applied to a single tendon, and a subsequent *X*-axis concentrated load of 1.1N to emulate complex operational scenarios.

**Figure 6:**
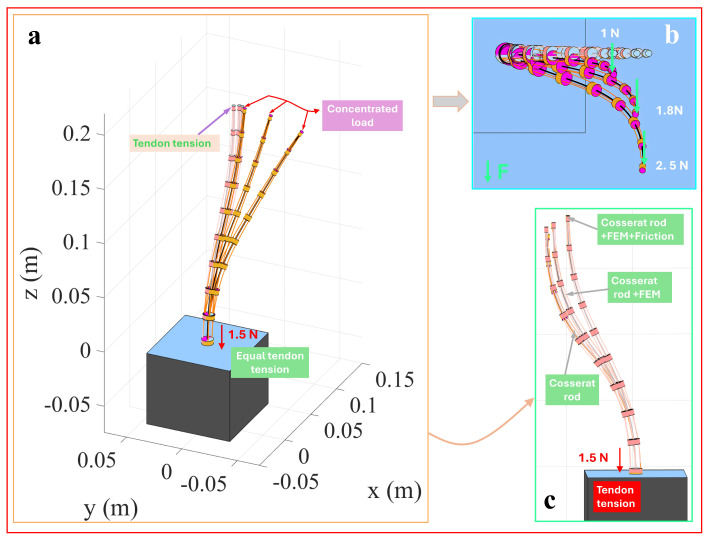
Converging tendon-routing TDCR with concentrated load.

Figure 6a illustrates the overall deformation characteristics under a 1.5N tendon force and concentrated terminal load, showing increased bending of the TDCR as the concentrated load was increased from 1.0N to 2.5N. Figure 6b provides a top view, revealing nonlinear bending near the disk caused by the coupling effect of friction and localized loading. Figure 6c compares the deformation differences under identical tendon forces using the classical Cosserat rod, discrete concentrated force, and friction-inclusive discrete concentrated force models. The results indicate that concentrated loads have a pronounced effect on local path distortion in the convergent tendon routing, providing theoretical support for designing and optimizing tendon-driven systems.

A comprehensive evaluation of the simulation results for four tendon-routing (single-segment/parallel, two-segment, helical, and convergent) and three modeling approaches (classical Cosserat rod continuous force, discrete concentrated force, and friction-inclusive discrete concentrated force) reveals consistent trends. Regardless of the routing or segment configuration, the classical Cosserat rod model predicts the most significant overall deformation, followed by the discrete concentrated force model, while the friction-inclusive discrete concentrated force model exhibits significantly reduced deformation predictions. The helical routing demonstrates the ability to achieve high degrees of spatial deformation using a single wound tendon without requiring additional actuation sources. In contrast, the convergent routing offers advantages in end-effector size control but exhibits more pronounced local friction and nonlinear effects. Comparative analyses were conducted under varying external concentrated loads to investigate the coupling effects of tendon force and external loads further. However, these findings remain at the numerical simulation stage. Experimental validation is required to confirm these conclusions’ effectiveness and practical applicability.

### 3.2. Experimental System

While the aforementioned numerical simulations clarified the differences between various tendon-routing and mechanical models, their practical feasibility and reliability require further experimental validation. To this end, the experiments used the friction-inclusive discrete concentrated force model as the primary reference. Consistent loading conditions were applied to a physical prototype to compare the experimental measurements with the numerical predictions, thereby assessing the accuracy and applicability of the proposed model.

**Figure 7:**
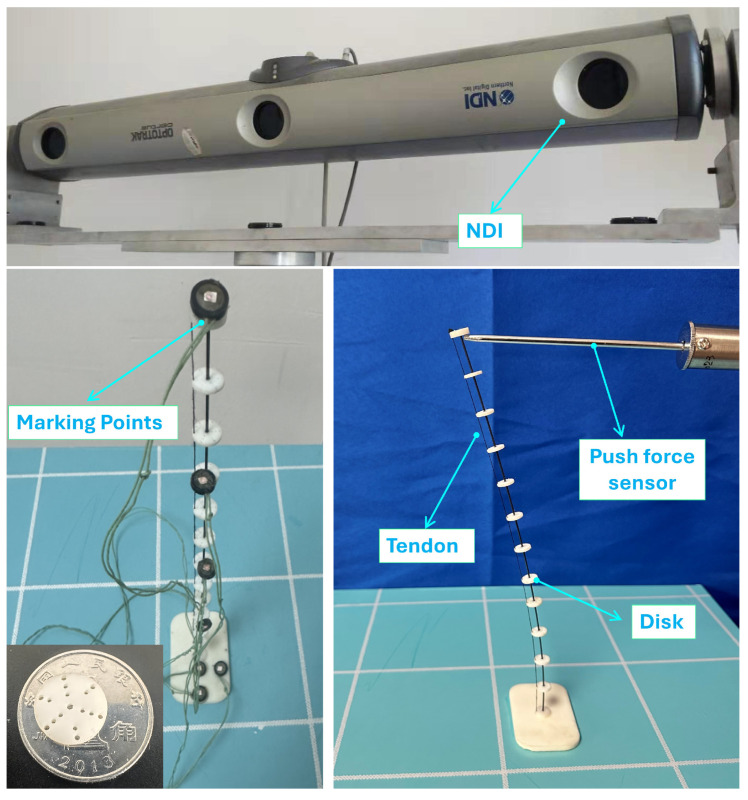
TDCR experimental platform.

Figure 7 illustrates the prototype device used in this study. The device consisted of a nickel-titanium alloy rod with a diameter of d=0.9mm and a length of l=250mm. Along the backbone, 13 evenly spaced disks were fixed with a diameter of 10mm. These disks were fabricated from polyamide material using 3D printing and bonded to the central rod using 502 adhesive. The backbone, a nickel-titanium alloy rod, was supplied by Wuxi Puruite Technology, China, with a diameter of 0.9mm. The tendons were made of acrylic fiber lines with a diameter of 0.4mm, and the offset distance from the tendon hole to the central hole was 0.43mm. The tendons were arranged on the backbone in specific configurations depending on the experimental conditions.

To achieve precise three-dimensional spatial position and orientation measurements, the NDI (The device was manufactured by Northern Digital Inc. in Radolfzell, Germany). Optotrak Certus optical measurement system was employed, as shown in Figure 7. This system uses infrared cameras to capture the spatial coordinates of markers, enabling real-time, non-contact spatial tracking of objects. During the experiments, the NDI probe was attached to key points on the tendon-driven continuum robot to ensure accurate measurements of endpoint deflection and bending angles under various loading conditions. The specific system parameters used in this study included a sampling frequency of 100Hz, a spatial resolution of 0.01mm, and a measurement error of less than 0.1mm, meeting the high-precision tracking requirements of this experiment.

### 3.3. Experimental Procedures

To further validate the accuracy and applicability of the numerical predictions regarding the deformation characteristics of the TDCR, this section introduces the overall approach and key steps of the experimental tests. By conducting multi-perspective measurements and recordings of the TDCR’s structure, mass, and applied loading conditions, essential parameters such as the stiffness matrix were extracted and aligned with the simulation input conditions, enabling a direct comparison between the experimental and simulation results. The specific experimental steps are summarized as follows:

Step 1: The mass of the TDCR was measured using an electronic balance. This measurement included the mass of the backbone, the individual disks, and the total mass of the assembled disks on the backbone. The backbone with the attached disks was then fixed to a base, and its reference state was recorded. Various thrust forces or weights were applied to the TDCR’s distal end, and the NDI system was used to measure the deformation and bending positions of the central rod. The measured deformations under different applied forces were used to calculate the stiffness matrix of the TDCR theoretically. This stiffness matrix served as an input parameter for the simulations.

Step 2: The tendons were arranged on the TDCR in various configurations according to different experimental conditions. At the actuation end, the tendons passed over fixed pulleys, and different weights were suspended at the pulley ends to achieve varying tendon driving forces.

Step 3: Concentrated loads were applied to the TDCR, or different weights were used as the tendon driving forces. The positional information of the backbone was captured using the NDI system.

Step 4: The stiffness matrix identified through the imaging system was set as a parameter for the simulation. Using the data measured by the NDI system, the shape of the TDCR backbone was reconstructed through interpolation and compared with the simulated experimental data.

### 3.4. TDCR Parameter Identification

The TDCR prototype is shown in Figure 7. Due to the use of adhesive to fix the disks onto the backbone, the continuous stiffness varies. To ensure consistency between the simulation parameters and the actual model, the accurate stiffness matrix of the robotic system was required and obtained through system identification methods. Under quasi-static conditions, the TDCR system typically satisfies the following relationship:

(36) $$f_{meas}=Ku$$

where $f_{meas}\in R^{n}$ represents the measured external force vector, $u\in R^{n}$ denotes the measured displacement vector, and $K\in R^{n\times n}$ is the stiffness matrix to be identified. Through multiple loading experiments, *m* sets of force data $f_{meas,i}$ and displacement data $u_{i}$ were obtained. By stacking these *m* data sets, the overall system equation is expressed as:

(37) $$F_{meas}=\left[\begin{array}{c} f_{meas,1}\\ f_{meas,2}\\ \vdots \\ f_{meas,m} \end{array}\right]=\left[\begin{array}{c} u_{1}\\ u_{2}\\ \vdots \\ u_{m} \end{array}\right]K=UK$$

where $F_{meas}\in R^{m\times n}$ represents the measured force matrix, and $U\in R^{m\times n}$ denotes the displacement matrix. To obtain the optimal solution under experimental errors and noise, the least squares method is applied:

(38) $$\min_{K}{∥F_{meas}-UK∥}_{F}^{2}$$

where ${∥\cdot ∥}_{F}$ represents the Frobenius norm. To prevent ill-posed problems and overfitting, a regularization term ${\lambda ∥K∥}_{F}^{2}$ is added, where λ is the regularization parameter. The optimization problem is then reformulated as follows:

(39) $$\min_{K}∥F_{meas}{-UK∥}_{F}^{2}+\lambda {∥K∥}_{F}^{2}$$

To compare the predicted displacements with the actual measurement results and validate the accuracy of the stiffness matrix, the identified stiffness matrix K is used to predict the displacement $u_{pred}$ under a new external force $f_{ne}$. The validation formula is expressed as follows:

(40) $$u_{pred}=K^{-1}f_{ne}$$

### 3.5. Single-Segment Collinear Tendon Routing

This study conducted experimental research on a single-segment TDCR with collinear tendons to investigate the impact of parallel tendon configurations on deformation characteristics under different tension conditions. Five tendon tension schemes were designed (ranging from 0.5N to 2.5N), and a fixed concentrated load of 0.05N was applied under each tension condition to quantify the endpoint displacement and bending angles. The primary objective was to evaluate the predictive accuracy of the simulation model under the coupled effects of different tendon tensions and concentrated loads, thereby providing data support and theoretical guidance for optimizing TDCR design.

**Figure 8:**
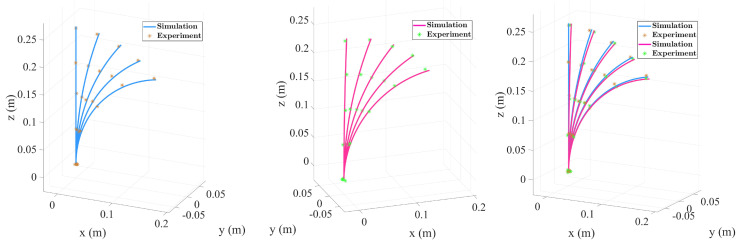
Experimental and simulation results of single-segment TDCR.

The experimental and simulation results are compared in Table 1, with data visualizations presented in Figure 8. Figure 8 illustrates the bending shape induced solely by tendon tension without external concentrated loads, while Figure 8 depicts the bending shape after applying a 0.05N concentrated load at the end. Figure 8 overlays the two conditions for direct comparison. Under no-load conditions, the experimental data (marked with asterisks) showed a maximum deviation of approximately 0.2% from the simulation results (curves), indicating the high predictive accuracy of the simulation model under low-load scenarios. However, after applying the concentrated load, the experimental results revealed a significant increase in the bending angle, with the deviation between the simulation and experiment rising to 0.25%. This increased deviation was primarily attributed to the amplifying effect of external loads on tendon tension, material nonlinearity, and the greater significance of frictional forces at higher tensions.

Further analysis revealed that the terminal concentrated load is directly coupled with tendon tension and interacts with the tendon actuation method. In parallel routing, where all tendons are distributed on the same cross-section, tension and concentrated loads are more likely to create additive effects on the backbone, significantly increasing bending. Specifically, under the five tendon tension conditions, the end displacement increased from 3.7mm at the lowest tension of 0.5N to 143.6mm at the highest tension of 2.5N while the bending angle rose from 3.91° to 37.29°. The amplifying effect of higher tension on end deformation was particularly pronounced.

Notably, although some deviations between the simulation and experimental results were observed under high-tension conditions, the model maintained good agreement with the experiments within this range, with deviations not exceeding 0.25%. This demonstrates that the current model exhibits high applicability under both low-load and high-load scenarios for parallel tendon routing. It provides valuable data references for guiding the design of multi-degree-of-freedom or multi-segment structures. However, these results also highlight potential sources of deviation under more extreme conditions, such as higher tension or more complex curvatures. These include the coupling effects of external loads with material nonlinearity and the increased friction on tendon contact surfaces as tension rises.

### 3.6. Two-Segment Collinear Tendon Routing

**Figure 9:**
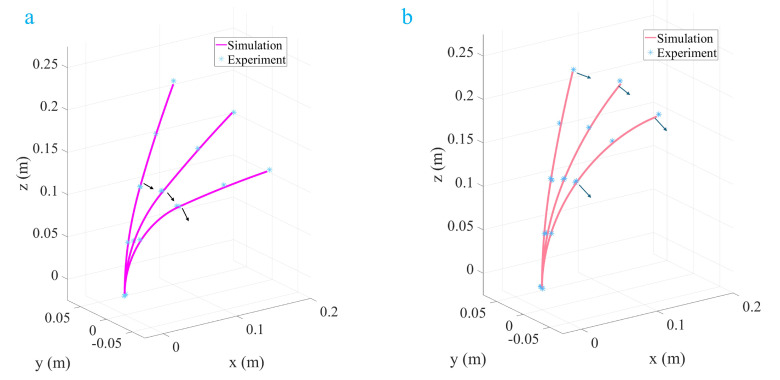
Experimental and simulation results of two-segment parallel routing TDCR.

This study conducted experimental research on a two-segment TDCR with collinear tendons to investigate the coupling effects between different tension combinations, concentrated loads, and end deformation and the system’s compliance. This study also aimed to evaluate the accuracy and applicability of the simulation model. Table 2 summarizes the experimental data, and deformation visualizations under various conditions are shown in Figure 9. Figure 9a illustrates the bending shape under actuation by the first segment’s tendons without concentrated loads. The maximum deviation between the experimental measurements (marked with asterisks) and simulation predictions (curves) was 0.16%, indicating that the model accurately captured the bending behavior of the two-segment structure under low-load conditions. However, when a concentrated load was applied to the second segment (Figure 9b), the end bending angle increased significantly, and the maximum deviation rose to 0.3%.

This increase in deviation could be attributed to several factors. First, higher tension levels amplified the frictional forces between the tendons and the backbone. Second, the nonlinear stress–strain characteristics of the material became more pronounced under high loads. Lastly, simplifications in the simulation, such as assumptions regarding the inter-segment connection structure and local contact effects, introduced discrepancies between the actual force distribution and theoretical analysis. These factors became increasingly coupled under high-tension or large-deformation conditions, leading to more significant divergence between the experimental and simulation results.

Further analysis revealed that concentrated loads in the two-segment structure shifted the bending center position at the end and regulated the overall compliance. Under certain high-tension combinations, concentrated loads significantly increased the bending curvature. For example, when the tension in the first segment was 5N and the second segment was 2N, the end displacement increased from 67.4mm to 148.1mm, and the bending angle rose from 15.75° to 38.86°. This phenomenon can be interpreted as a “load amplification” effect, where external concentrated loads enhance bending in specific segments of the coupled flexible structure, providing the TDCR with greater adaptability for confined spaces and complex path operations.

However, this amplification effect also increases the influence of friction and material nonlinearity on predictive accuracy. Further calibration or refinement of the model could involve quantifying friction coefficients and local contact stiffness through additional experiments for applications requiring higher precision. Overall, this study found that the deformation response of the two-segment TDCR under combined high tension and concentrated loads is more flexible, although deviations tend to increase. Nevertheless, the overall deviation level, with a maximum of approximately 0.3%, remains within an acceptable range.

### 3.7. Helical Tendon Routing

This study proposed a helical tendon routing for a two-segment TDCR, aimed at enhancing its spatial deformation capacity and load-bearing performance without increasing the system weight. Utilizing a 720° winding path, the helical tendons created more complex force patterns, allowing the TDCR to maintain excellent bending capability and structural stiffness under high-tension conditions.

**Figure 10:**
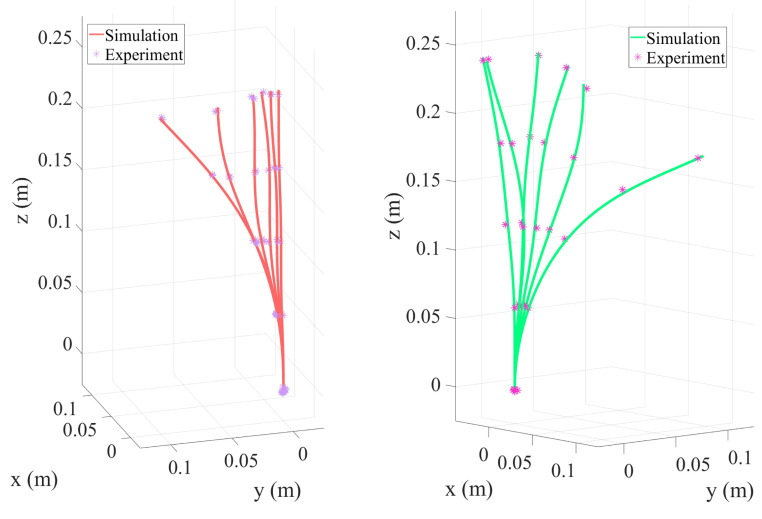
Experimental and simulation results of TDCR with helical tendon routing.

As shown in Figure 10 and Table 3, increasing the tendon tension from 5N to 30N resulted in gradual increases in the end displacement and bending angle under no-load conditions. When a concentrated load was applied, the curvature amplification effect became pronounced in the high-tension range, with the bending angle increasing from 24.79° under no load to 39.06° under an applied load. This demonstrates helical routing’s exceptional ability to adjust compliance, making it particularly suitable for operations in confined spaces and along complex paths.

Compared to parallel or collinear tendons, the helical routing advantages primarily arise from its more extended effective moment arm and broader backbone–tendon contact area. Without adding extra mass or independent drive tendons, the twisting–bending coupling effect induced by the winding path enhances structural stiffness and maintains controllability under high-load conditions. Furthermore, the experimental and simulation results indicated that the deformation amplitude gradually grew under slowly applied loads. However, the experiments also revealed a bifurcation phenomenon: once external forces exceeded a critical threshold, the system exhibited a rapid nonlinear deformation response, likely due to the combined effects of local friction and material nonlinearity in high-stress regions.

### 3.8. Converging Tendon Routing

**Figure 11:**
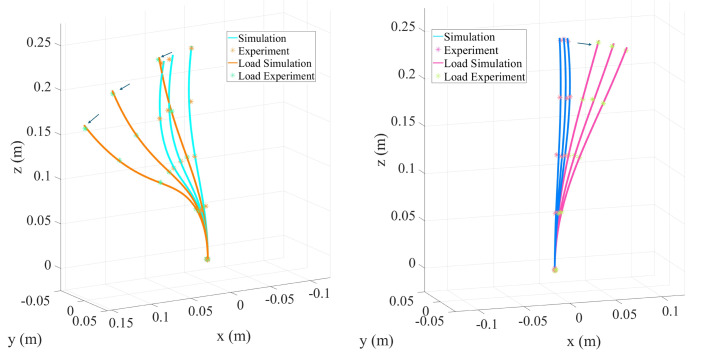
Simulation and experimental results of convergent tendon routing.

This study focuses on the numerical simulation and experimental comparison of the convergent tendon routing in a TDCR, aiming to explore its performance potential in scenarios with narrow spaces and high compliance requirements. Compared to other tendon routing, such as parallel or helical, convergent routing gradually gathers multiple tendons into a smaller diameter, making the distal end more compact. This configuration is advantageous for applications with strict spatial constraints, such as minimally invasive surgeries and endoscopic procedures. Figure 11 and Table 4 show the end displacement and bending angle of the TDCR under different tendon tensions (ranging from 5 N to 40 N) and concentrated loading conditions. Under no load, the end displacement increased from 19.4 mm to 57.1 mm. When a concentrated load was applied, the displacement surged to 157.8 mm, and the bending angle increased from 13.65° to 41.81°. The experimental data (denoted by asterisks) and simulation results (represented by curves) showed a high degree of agreement, demonstrating the convergent routing ability to balance considerable bending and a compact distal end design effectively.

Although the average deviation between the experimental and simulation results under high-load conditions was only 0.26%, it should be noted that the assumption of linear materials and the neglect of friction effects may lead to an underestimation of the end deformation in the simulations. At high tensions, both the backbone and tendon materials are more likely to exhibit nonlinear stress–strain behavior, and frictional forces, which increase with contact pressure, amplify the effect on the end pose. Particularly in convergent routing, where tendons are more concentrated at the distal end, and the local loading is coupled, this further amplifies the sources of error. To improve the model’s accuracy, future work could incorporate numerical corrections based on nonlinear materials or multi-contact friction models and calibrate the model by experimentally measuring the friction coefficient and the backbone’s elastic curve. This would enable more accurate predictions of TDCR deformation behavior in high-load environments.

## 4. Discussion

A more physically realistic TDCR mechanical model was developed by explicitly incorporating friction effects and discrete tendon concentrated force actuation into the Cosserat rod theory. Its accuracy and applicability under various loading conditions were validated through numerical simulations and experimental comparisons involving four tendon-routing configurations: single-segment parallel, two-segment parallel, helical, and convergent. The comprehensive results demonstrated that, for relatively low tendon tensions (0.5–2.5 N) and small concentrated loads (0.05 N), the discrepancy between the simulations and experiments remained approximately within 0.2–0.25%. However, as the tension and load increased, material nonlinearity and frictional amplification at the tendon–backbone interface became more pronounced, particularly in multi-segment structures and in helical or convergent routing configurations, resulting in a maximum deviation of around 0.3%. This finding sharply contrasts with predictions from idealized Cosserat rod models, highlighting that neglecting friction and discrete force distribution can lead to underestimating bending amplitude and coupling effects under high-load conditions.

Further analysis of the discrepancies suggested three leading causes. First, significant nonlinear stress–strain relationships arose at high-stress levels, whereas small-strain assumptions were still employed for the backbone and tendons, impeding accurate characterization of extreme loading scenarios. Second, friction effects under high tension or curvature were amplified by increased contact pressure, causing substantial tip offset. This phenomenon was especially evident at the inter-segment connections in the two-segment structure, and it was intensified by torsion-bending coupling in the helical routing and by localized load channels in the convergent routing. Third, simplifying assumptions (such as neglecting disk thickness or ignoring higher-order dynamics and changes in friction coefficients) may have introduced cumulative errors under high-load conditions, producing more significant discrepancies between the simulation and experiment than those observed under moderate or low loads.

From a comparison of the four configurations, the parallel routing was found to be suitable for verifying whether distal bending behavior could be accurately reproduced under low- to moderate-load conditions. The primary difference between the single-segment and two-segment parallel approaches lies in the amplification of the bending center and compliance caused by inter-segment coupling. The helical routing, employing a 720° winding path, increased the load-bearing capacity and controllability without adding actuation sources or system weight; the experiments indicated that it maintained substantial bending and structural stability under tensions ranging from 5 N to 30 N. By converging the tendons at the distal end, the convergent routing reduced the outer diameter and thus showed potential for minimally invasive surgery or confined spaces. However, its sensitivity to material nonlinearity and friction was magnified under high-load conditions, potentially leading to significant errors during pronounced bending. Additional sensors should, therefore, be employed in future studies to measure contact friction and real-time mechanical responses.

Although this study introduced discrete concentrated force actuation and friction effects into the Cosserat rod model and validated these improvements experimentally, several limitations persist: (1) assuming negligible disk thickness may underestimate local stress concentration; (2) default friction modeling mainly addressed pulley-type friction and did not account for potential nonlinear variations in friction coefficients and normal loads under high curvature; and (3) evaluations of TDCR deformation were restricted to static or quasi-static conditions, leaving the applicability to complex dynamic scenarios unexamined.

## 5. Conclusions

In this study, we developed a discrete concentrated wrench-driven TDCR model based on Cosserat rod theory. This model emphasizes piecewise linear tendon segments and incorporates frictional interactions between the tendons and disk holes. Utilizing this framework, we evaluated multiple tendon-routing schemes. Compared to conventional continuous force-driven Cosserat rod-based models, the discrete approach more accurately mirrors real-world mechanics, enabling more precise predictions of key parameters such as tendon tension and posture during flexible manipulation.

Both numerical simulations and experimental studies demonstrated that accounting for friction effects and employing discrete concentrated force modeling significantly enhance the accuracy of posture control and load prediction. Notably, when friction was considered, the average maximum deviation across different tendon-routing schemes was reduced to merely 0.3%, underscoring the effectiveness of our approach.

Despite constructing a more realistic model by incorporating friction, further investigations are necessary to evaluate the model’s applicability in various medical scenarios. Future research should explore variations in tendon materials, disk thicknesses, and dynamic friction ranges to ensure comprehensive applicability. Additionally, integrating this model with advanced control algorithms will be essential to support its practical implementation in specific clinical contexts.

## Figures and Tables

**Table 1:** Deformation results of single-segment TDCR under concentrated load.

Tension (N)	End Offset (mm)	End Bending Angle (°)	Load (N)	End Bending Angle Under Load (°)
0.5	3.7	3.91	0.05	5.61
1	46.7	10.76	0.05	12.22
1.5	89.3	19.74	0.05	20.10
2	111.7	27.50	0.05	28.73
2.5	143.6	37.29	0.05	41.18

**Table 2:** Experimental deformation results of two-segment parallel tendon routing.

Segment 1	Segment 2	End Offset (mm)		End Bending Angle (°)		Load (N)	
Tension (N)	Tension (N)	Segment 1	Segment 2	Segment 1	Segment 2	Concentrated Load	End Load
5	2	17.8	67.4	8.27	15.75	1	0
16	2	49.2	148.1	24.15	38.86	1	0
27	2	72.4	196.3	38.31	61.50	1	0
2	2	11.7	41.3	5.52	9.63	0.6	0.5
2	8	27.9	104.7	13.03	25.75	0.6	0.5
2	18	47.4	154.2	22.75	42.56	0.6	0.5

**Table 3:** Comparison of experimental results under helical tendon routing.

Tendon Tension (N)	End Concentrated Load (N)	End Y-Axis Offset (mm)		End Bending Angle (°)	
Applied Force	Applied Load	Without Load	With Load	Without Load	With Load
5	0.8	4.3	20.7	0.99	5.50
10	0.8	10.8	37.4	2.48	9.43
15	0.8	16.3	17.3	3.86	10.25
20	0.8	24.6	17.2	5.69	4.89
25	0.8	53.7	100.6	12.66	26.67
30	0.8	86.2	85.4	24.79	39.06

**Table 4:** Experimental results of convergent tendon routing.

Tendon Force (N)	End Offset in x-Direction (mm)		End Bending Angle in x-Direction (°)		End Load (N)
	Without Load	With Load	Without Load	With Load	
5	19.4	61.5	4.47	14.36	0.5
20	44.8	122.9	10.58	30.19	0.5
40	57.1	157.8	13.65	41.81	0.5
1	5.4	47.8	1.29	11.07	1
2	10.5	66	2.42	15.36	1
3	13.3	81.2	3.05	19.21	1

## Data Availability

The data are contained within this article.
